# Inhibition of ALDH2 by quercetin glucuronide suggests a new hypothesis to explain red wine headaches

**DOI:** 10.1038/s41598-023-46203-y

**Published:** 2023-11-20

**Authors:** Apramita Devi, Morris Levin, Andrew L. Waterhouse

**Affiliations:** 1 Department of Viticulture and Enology, University of California, Davis, CA USA; 2 Department of Neurology, University of California, San Francisco, CA USA

**Keywords:** Headache, Enzyme mechanisms, Oxidoreductases, Mechanism of action, Neuroscience, Neurology

## Abstract

The consumption of red wine induces headaches in some subjects who can drink other alcoholic beverages without suffering. The cause for this effect has been attributed to a number of components, often the high level of phenolics in red wine, but a mechanism has been elusive. Some alcohol consumers exhibit flushing and experience headaches, and this is attributed to a dysfunctional ALDH2 variant, the enzyme that metabolizes acetaldehyde, allowing it to accumulate. Red wine contains much higher levels of quercetin and its glycosides than white wine or other alcoholic beverages. We show that quercetin-3-glucuronide, a typical circulating quercetin metabolite, inhibits ALDH2 with an IC_50_ of 9.6 µM. Consumption of red wine has been reported to result in comparable levels in circulation. Thus, we propose that quercetin-3-glucoronide, derived from the various forms of quercetin in red wines inhibits ALDH2, resulting in elevated acetaldehyde levels, and the subsequent appearance of headaches in susceptible subjects. Human-subject testing is needed to test this hypothesis.

## Introduction

Headache is a common affliction, affecting about 16% of the world’s population daily^1^. The major headaches are primary headaches, namely tension-type headaches, cluster headaches, and migraine. Headaches, particularly migraine attacks, are a significant cause of disability. Migraine remains second among the world’s cause of disability, and first among young women^2^.

Alcoholic beverages (beer, wine, spirits) are the most common dietary agents associated with headaches, with at least occasional triggering in 37% of patients^3,4^. Alcoholic beverages are associated with two types of alcohol-induced headaches, per the International Headache Society (IHS). First, immediate, or primary headache (8.1.4.1 of the International Classification of Headache Disorders [ICHD]-III) initiates within 3 h of alcohol ingestion and resolves within 72 h after alcohol ingestion stops. Second, delayed alcohol-induced or hangover headache (8.1.4.2 of the ICHD-III beta) developed within 5–12 h of alcohol ingestion and resolves within 72 h^4,5^.

Alcohol is known to induce headaches when consumed in large quantities. The alcohol induced headaches have variously been ascribed to either the direct effect of alcohols, metabolism of alcohol, genetic makeup, and presence of congeners^6^. Alcohol is metabolized in the liver to acetate in a two-step process: alcohol (ethanol) is converted to acetaldehyde by alcohol dehydrogenase (ADH) followed by conversion of acetaldehyde to acetate by aldehyde dehydrogenase (ALDH). At higher ethanol concentration, there is a rapid conversion of ethanol resulting in acetaldehyde built up. Acetaldehyde can produce adverse effects such as nausea, diaphoresis, facial blushing, and headache at higher concentrations^6,7^. In fact, drugs such as disulfiram which inhibit aldehyde dehydrogenase (ALDH), and cause acetaldehyde accumulation if alcohol is consumed, are used as a treatment for alcoholism by causing discomfort noted above, including headache^8^ to discourage consumption. ALDH enzymes have several isoforms, thus have varying affinities for the substrates. The cytosolic ALDH1 and mitochondrial ALDH2 isoforms are the most important in acetaldehyde metabolism to acetate. ALDH1 has a low K_m_ (about 30 µM) whereas ALDH2 has a high K_m_ (0.2 µM) for acetaldehyde. Thus, ALDH2 rapidly eliminates acetaldehyde, maintaining 3 µM or lower concentrations in the bloodstream, roughly 1000-fold less than the levels in the liver^9^. There are two isoforms of ALDH2 enzyme: ALDH2*1 which is most common in most of the world’s population and a dysfunctional variant ALDH2*2, in approx. 40% of East Asians including Han Chinese, Japanese and Koreans^10^. No ALDH2 activity is exhibited in ALDH2*2 homozygotes whereas heterozygotes report a reduced activity of the enzyme. Most studies correlating low alcoholism among Asians with ALDH2*2 report a considerably higher blood acetaldehyde concentrations (30 to 75 µM or higher, 10 times higher than the normal levels) after alcohol consumption^9–12^. This high level of acetaldehyde causes facial flushing, headache, tachycardia, and nausea, similar to disulfiram treatment^9^. This similarity between these two scenarios and the accumulation of acetaldehyde suggests a relationship between acetaldehyde accumulation and headache caused by alcoholic beverages.

In a meta-analytic review on alcohol use disorders (AUDs) in primary headache, 28% of studies endorsed red wine as the trigger, followed by spirits (14%), white wine (10%) and sparkling wine/beer (10%)^13^. Red wine headache (RWH) does not require excessive amounts of wine as a trigger. In most cases, the headache is induced in 30 min to 3 h after drinking only one or two glasses of wine^3^. Wine constituents such as biogenic amines, sulphites, phenolic flavonoids, or tannins have been reported as the possible cause of wine headaches^3,4,14–18^. Nevertheless, no chemical constituent has been clearly implicated as the primary trigger of red wine headache (RWH) nor has a mechanism for eliciting the headache been proposed.

The higher amount of phenolic compounds, especially flavonoids, in red wine, tenfold compared to white wine, make them a primary contender responsible for RWH^16^. However, phenolics and high phenolic foods have not been linked to headache. Interestingly, some red wine phenolics such as quercetin, and resveratrol have been reported to affect the activity of ALDH^19,20^. Quercetin was reported as potent inhibitor of cytosolic aldehyde dehydrogenase (ALDH1) at low concentration (< 1 mM) of acetaldehyde and cofactor NAD+^19^. However, these studies were conducted to relate the activity of the ALDH with anticancer properties and birth defects^19,21^ and not with acetaldehyde metabolism. Further, none of the mentioned studies reported the effect of quercetin on mitochondrial aldehyde dehydrogenase (ALDH2). A study by Keung and Vallee^22^ reported the effect of several flavonoids including quercetin on both ALDH1 and ALDH2 activity. They found no effect of quercetin and other red wine flavonoids (kaempferol, rutin and myricetin) on both aldehyde dehydrogenases. But the enzyme assay in the study was performed at pH 9.5, and thus are of marginal significance at physiological pH. In another study Orozco et al.^23^ reported inhibition of yeast cytosolic and mitochondrial ALDH by quercetin during red wine fermentation. Thus, evaluation of red wine flavonoids on ALDH2 activity, with possible effect on acetaldehyde metabolism might provide clues to RWH.

In order to assess this possible mechanism for RWH, we evaluated the inhibition of mitochondrial ALDH2 by red wine flavonoids, especially quercetin derivatives, using an in vitro enzymatic assay.

## Results

### In vitro inhibitory effect of red wine phenolics/flavonoids on ALDH2

The inhibitory effect of selected wine phenolics/flavonoids (at 20 µM concentration) was evaluated in vitro on the inhibition of ALDH2 (in %, Table 1). Among the selected compounds, quercetin glucuronide (compound 9), a liver metabolite of quercetin, showed the highest inhibitory activity (78.69 ± 1.21%) whereas the least inhibition was observed for epicatechin (0.34 ± 0.12%). For all other compounds (1–8, 10–12), the inhibition activity ranged from 14.77 ± 0.39 to 27.69 ± 0.61%, suggesting a low to moderate inhibition effect of wine flavonoids on ALDH2.

**Table 1:** Inhibition of ALDH2 activity by selected red wine phenolics using the QuantiChrom™ kit.

S.no.	Compounds (20 μM)	ALDH2 inhibition (%)
1	Quercetin dihydrate	25.71 ± 2.19^f^
2	Quercetin	27.69 ± 0.61^f^
3	Quercetin glucoside	17.56 ± 1.84^b,c,d^
4	Quercetin galactoside	20.61 ± 0.79^d,e^
5	Quercetin-3-rhamnoside	21.46 ± 1.90^e^
6	Quercetin-7-rhamnoside	19.58 ± 0.32^d,e^
7	Rutin	18.53 ± 0.06^c,d,e^
8	Tamarixetin	25.83 ± 1.31^f^
9	Quercetin glucuronide	78.69 ± 1.21^g^
10	Kaempferol	15.62 ± 0.78^b,c^
11	Myricetin	21.78 ± 1.56^e^
12	Catechin	14.77 ± 0.39^b^
13	Epicatechin	0.34 ± 0.12^a^

Values are mean ± SD for n = 3 for each phenolic compound.

Different alphabet in superscript represent that the values are significantly different at p < 0.05.

Further, it was observed that glycosylated forms of quercetin (compounds 3–7) exhibited lower inhibitory activity than the aglycone (compounds 1–2). However, the methoxylated quercetin, tamarixetin did not differentially inhibit the enzyme compared to quercetin.

**Figure 1:**
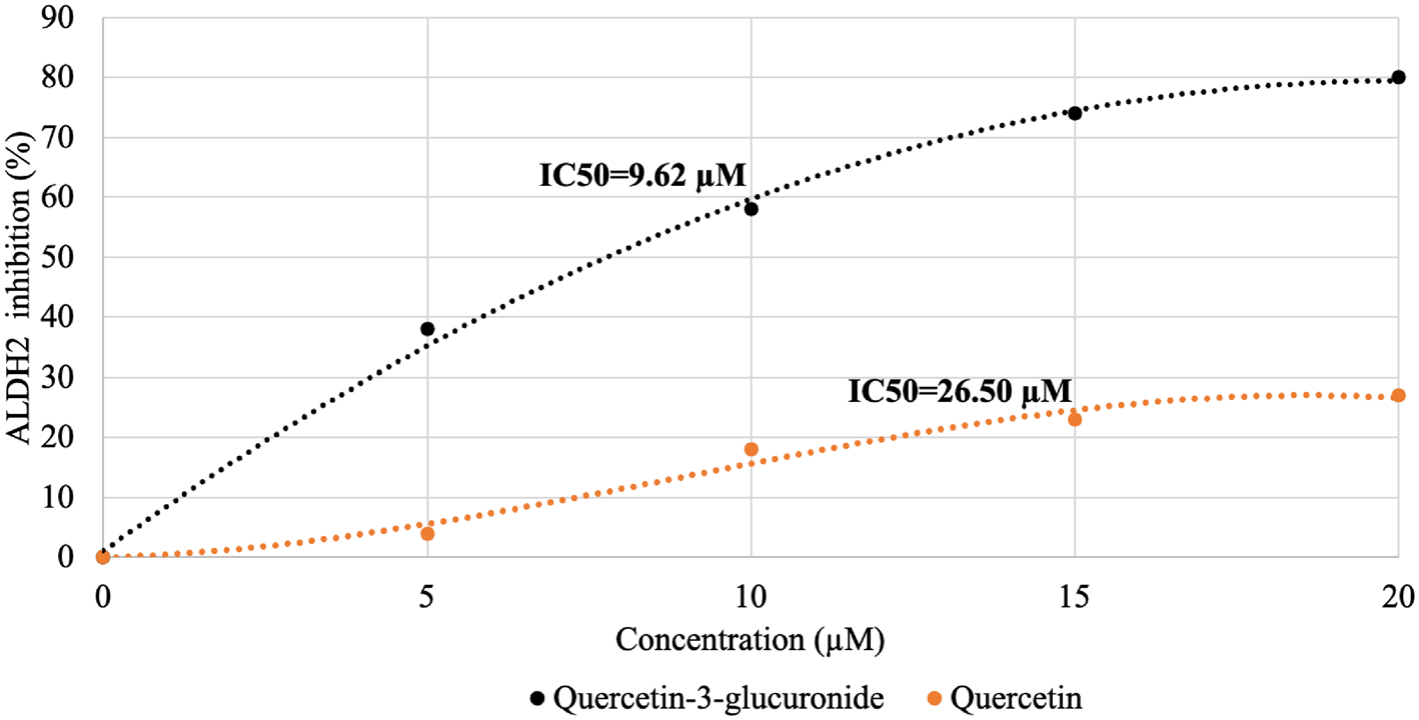
ALDH2 inhibition at various concentrations of quercetin-3-glucuronide and quercetin.

Quercetin-3-glucuronide was selected as a potent inhibitor for further study based on the inhibition screening assay. The ALDH2 inhibition by different concentrations of quercetin-3-glucuronide (5–20 µM) is depicted in Fig. 1. Quercetin-3-glucuronide has an IC_50_ of 9.62 µM, a stronger inhibition of ALDH2 than the aglycone, quercetin (IC_50_ of 26.50 µM). However, the inhibition by quercetin-3-glucuronide is lower than the drug disulfiram (IC_50_ of 1.45 µM, data not shown), used clinically to inhibit the enzyme.

## Discussion and conclusion

Flavonols, such as quercetin, myricetin, and kaempferol, are present in wine, either as glycosides or the aglycone. In two studies, the total flavonol content in white wines (mean value: traces to 7 mg/L) are almost ten-fold lower than the red wines (mean value: 4–93 mg/L)^24,25^. Burns et al.^26^ reported total quercetin and free quercetin ranging from 5 to 104.7 µmoles and 1.8–41.9 µmoles in 16 red wines from different wine countries, varieties and vinification methods. Many other studies have reported quercetin contents in Italian^27,28^, Australian^24^, Spanish^29^, and Bulgarian^30^ wines. It is evident from the reports that there is a variation in the quercetin content in the red wines, and most investigations evaluated a relatively small number of samples (n < 20).

Quercetin-3-glucuronide is also reported in red wines as one of the major quercetin glycosides^24,31,32^. Castillo-Muñoz et al.^32^ found quercetin-3 glucuronide in the range of 10.26 to 13.81 mg/L in different *Vitis vinifera* single cultivar red wines. Ghiselli et al.^31^ observed 19 mg/L of quercetin-3-glucuronide in Italian Sangiovese wine. Jeffery et al.^24^ found quercetin-3-glucuronide as the predominant and most stable glycoside, ranging from below detection level to 39.67 mg/L among 121 Australian red wines.

Studies have repeatedly noted that the level of quercetin is 4 to 8 times higher in sun exposed grape clusters than the shaded clusters^33,34^. Ritchey and Waterhouse^35^ evaluated the total quercetin content in high-volume versus ultra-premium commercial Cabernet Sauvignon wines. The average total flavonols was four times higher in ultra-premium wines (202 mg/L) than in high-volume wines (53 mg/L). The study explained that vineyard practices (trellised vines, crop thinning, leaf removal) in the areas that produce ultra-premium wines would result in more sun exposure, which in turn, allows higher production of quercetin. But, the variations in levels arise not just from differences in grape composition induced by sun exposure, but also from wine-making techniques, including skin contact during fermentation, stabilization/fining procedures, and aging methods^26^. Quercetin absorption pathways in the gastrointestinal tract of humans and mammals are well understood^36^. Most quercetin absorption occurs in the small intestine, and a minor amount is absorbed in the stomach^36^. Quercetin aglycone and glucosides are not found in blood plasma upon absorption. In plasma, it appears as conjugates (78–79%), including quercetin-3-glucuronide, quercetin-3-sulfate, and methylated forms such as tamarixetin (10–13%) and isorhamnetin (8.5 to 11%)^36,37^. Numerous studies have been reported on quercetin bioavailability from foods and food extracts (onions, blackcurrants, bilberries, apples, red grapes, tomatoes), beverages (tea, fruit juices, coffee, cocoa, and wine) and supplements in the form of a solution, powder, capsule, or tablet^36^. The quercetin derivatives or catabolites are generally measured in blood and urine to assess their bioavailability. de Vries et al.^38^ compared the bioavailability of flavonol from 750 mL red wine (47.0 µmole quercetin), 50 g fried onion (52.6 µmole quercetin), and 375 mL black tea (45.3 µmole quercetin) in 12 healthy men. They observed that with comparable “doses” the plasma quercetin (including conjugates) level after red wine consumption (0.026 µM) is half of that of onions (0.053 µM). The study also found variation in plasma quercetin levels within persons of 10% and between persons of 10–20%. In another study, Spaak et al.^39^, the consumption of 2 drinks of Pinot noir containing 37.7 µmole of quercetin resulted in a blood plasma total quercetin concentration of 2.74 µM. Goldberg et al.^40^ obtained a blood plasma total quercetin level of 4.19 µM after consumption of white wine that contained a dose of 25 mg/70 kg body weight quercetin aglycone. The total concentration of quercetin and conjugates detected in blood plasma was 0.84 µM, i.e., 26.9% of the quercetin dose.

Hence, assuming the reported concentration of quercetin in wine as 346 µM, a standard drink of wine (147 mL) would have quercetin content of 50.6 µM. Thus, the blood plasma total quercetin concentration would be expected to be about 6 µM. Further, considering nearly 80% of the total quercetin in plasma corresponds to conjugated quercetin (mostly quercetin-3-glucuronide), the level in plasma would be 5 µM. Hence, based on our study, one standard drink of wine with 5 µM quercetin-3-glucuronide would result in nearly 37% of ALDH2 inhibition. The enzyme inhibition would result in acetaldehyde accumulation from the alcohol metabolism from wine, likely causing RWH, which otherwise is not evident in other foods (for instance, onions) with higher bioavailable quercetin but no alcohol. However, the actual inhibition will be higher or lower, depending on the concentration of other components, such as cofactors, buffers, etc. These results—suggest that RWH could be the result of ALDH2 inhibition by red wine quercetin metabolites.

Testing this hypothesis will require validation. An obvious experiment would be to compare wines having differing phenolic levels (particularly quercetin and total flavonols) with observed headache occurrences after ingestion, although there would be matrix differences. Controlling alcohol levels would be imperative. Another, simpler, experiment would be to provide RWH subjects with a quercetin supplement or placebo and a standard drink of vodka, to see if headaches result.

To probe the concept at a more fundamental level, other ideas come to mind. What concentration of circulating quercetin-3-glucuronide inhibit ALDH2 in vivo, yielding elevated levels of acetaldehyde? Are these levels correlated with the perception of a headache among RWH subjects? At the same concentrations, do RWH-susceptible subjects experience greater inhibition, and higher levels of acetaldehyde? Or, alternatively, do RWH subjects have a genetic tendency, to variations in ALDH2 isoforms (ALDH2*1 and ALDH2*2), to be more sensitive to acetaldehyde, and could this be related to the documented propensity of these subjects towards migraine headaches^3^. Also, genomic studies in these subjects could illuminate genetic polymorphism underlying this troublesome condition.

As noted above, there is considerable variation in quercetin levels among wines in the market. Hence, more detailed analysis of large samples of wines for quercetin levels could be helpful to guide wine drinkers who suffer RWH. But current measurement techniques are complex, so, a simple and fast detection method for flavanols or quercetin, such as a secondary spectral method, could provide a quick headache-potential assessment.

In conclusion, the present study provides an indication that the headache caused by red wine is due to the presence of quercetin and its glycosides, which upon metabolizing to quercetin glucuronide, inhibits ALDH2 enzyme activity. With the concurrent consumption of alcohol, the in vitro inhibition of ALDH2 by quercetin glucuronide would lead to an accumulation of toxic acetaldehyde, resulting in headaches. Further studies on human subjects are needed to verify this hypothesis.

## Materials and methods

### Chemicals and reagents

Quercetin, quercetin dihydrate, quercetin-7-rhamnoside, catechin and epicatechin were procured from Sigma-Aldrich, USA. Quercetin-3-glucoside, quercetin-3-galactoside, quercetin-3-rhamnoside, rutin (quercetin-3-rutinoside), tamarixetin (4-O-methyl quercetin), quercetin-3-glucuronide, kaempferol and myricetin were purchased from Extrasynthese, France. All the phenolics used are ≥ 99% purity (HPLC grade). Dimethyl formamide (DMF) from Sigma Aldrich, USA was used as organic solvent for preparing the phenolic compounds stock solutions (10 mM).

Human recombinant ALDH2 enzyme was purchased from Sigma-Aldrich, USA. The enzyme purity was > 90% and have the biological activity of > 0.14 units/mL as mentioned in the manufacturer’s certificate of analysis. The QuantiChrom™ aldehyde dehydrogenase inhibitor screening kit (EIAL-100) was procured from BioAssay Systems, Thermo-Fischer Scientific, USA for the quantitative determination of ALDH2 activity inhibition by phenolic compounds.

### Determination of ALDH2 inhibition by phenolic compounds

The ALDH2 inhibition by selected phenolic compounds was measured using the QuantiChrom™ aldehyde dehydrogenase inhibitor screening kit (EIAL-100, BioAssay systems, USA) as per the manufacturer’s instruction. In principle, the kit is based on enzymatic conversion of acetaldehyde to acetic acid and NADH by ALDH. The formed NADH in turn reduces a formazan reagent into a colored product, the absorbance of which measured at 565 nm, is proportional to the enzyme activity in the reaction. Briefly, the assay is performed in 96 well-plates by adding 0.1 U/mL of ALDH2, 20 µM phenolic compound in DMF to reaction mix comprising of assay buffer, NAD/MTT, diaphorase and 1× substrate provided in the kit. To the control, ALDH2 and solvent DMF without phenolic compound was added to the reaction mix as mentioned earlier. Blank reaction was prepared by adding 0.1 U/mL of ALDH2, solvent DMF without phenolic compound, assay buffer, NAD/MTT and diaphorase. The final volume of all the reactions was 100 µL. The reactions were incubated for 30 min at room temperature and the optical density was read at 565 nm (SpectraMax® iD3, Molecular Devices, USA).

ALDH inhibition for a test compound is calculated as follows:

$$\% inhibition=\left(1-\frac{\Delta\, OD\, test \,compound}{\Delta \,OD\, No\, inhibitor}\right)\times 100,$$

where ∆OD test compound is the OD_565nm_ value of test compound minus the OD_565nm_ value of blank well at 30 min.

∆OD no inhibitor is the OD_565 nm_ value of control minus the OD_565 nm_ value of blank well at 30 min.

### Statistical analysis

All the analyses were carried out in triplicates and reported as mean ± SD. The mean differences in the samples were assessed by Analysis of Variance (ANOVA) with post-hoc analysis using Tukey’s test at p < 0.05 using SPSS Statistics 29 software (IBM, USA).

## Data Availability

All data generated or analysed during this study are included in this published article.
